# The older sibling effect: comparing social functioning outcomes for autistic children with typically developing siblings, no siblings, and autistic siblings

**DOI:** 10.3389/fpsyg.2025.1568110

**Published:** 2025-03-31

**Authors:** Yonat Rum, Adi Dolev, Ofri Reichmann, Ditza A. Zachor, Einat Avni, Michal Ilan, Gal Meiri, Judah Koller

**Affiliations:** ^1^Seymour Fox School of Education, The Hebrew University of Jerusalem, Jerusalem, Israel; ^2^Faculty of Medicine, Tel Aviv University, Tel Aviv, Israel; ^3^The Autism Center/ALUT, Shamir (Assaf Harofeh) Medical Center, Zerifin, Israel; ^4^Azrieli National Centre for Autism and Neurodevelopment Research, Ben Gurion University of the Negev, Beer Sheva, Israel; ^5^Pre-School Psychiatry Unit, Soroka University Medical Center, Beer Sheva, Israel; ^6^Child and Adolescent Psychiatry, Soroka University Medical Center, Beer Sheva, Israel

**Keywords:** sibling, autism, autistic sibling, typically developing sibling, social functioning

## Abstract

**Background:**

Previous research showed that having older, typically developing (TD) siblings is associated with better social functioning in autistic children. Modeling by older siblings and the fact that siblings provide a social companion to practice social skills were suggested as explanations.

**Objectives:**

To investigate whether having older autistic siblings is associated with a similar or an opposite pattern.

**Methods:**

The Azrieli National Centre for Autism and Neurodevelopment Research in Israel database was used to retrieve data of autistic children who completed the Autism Diagnostic Observation Scale [ADOS-2] for secondary analyses. A cohort of 1,100 children was scanned to identify autistic children with no siblings (No-Sib; *n* = 146), older TD siblings (Older-TD-Sib; *n* = 300), and older autistic siblings (Older-Autistic-Sib; *n* = 40). Each Older-Autistic-Sib child was matched to (1) Older-TD-Sib, and (2) No-Sib, by sex, age, and cognitive scores, resulting in 29 triads of matched participants (*N* = 87). The three groups were compared on the ADOS-2 Social Affect sub-domain [a lower score (0–10) indicates less severe social-communication symptoms (better social functioning)].

**Results:**

Group comparisons revealed that autistic children with older TD siblings showed better social functioning than those with no siblings (*p* = 0.002, *adjusted p* = 0.007, d = 0.62). Autistic children with older autistic siblings showed a similar but weaker trend compared to those with no siblings (*p* = 0.082, *adjusted p* = 0.247, *d* = 0.40), and no difference was found between children with older TD versus autistic siblings (*p* = 0.647, *d* = −0.13; BF₀₁ = 4.55).

**Conclusion:**

Autistic children with autistic siblings demonstrated an “intermediate pattern,” implying a possible positive effect of having an older autistic sibling on social functioning, similar to that of having an older TD sibling, albeit smaller. This could be explained by complex relationships between sibling modeling and companionship or the impact of parenting measures, such as experience. These speculative explanations should be directly examined in future research.

## 1 Introduction

The sibling relationship is a meaningful connection in an individual’s life, previously found to influence development and psychological outcomes (Brody, 2004; McHale et al., 2012; Noller, 2005). Growing up with siblings offers children exposure to social-cognitive growth contexts, such as shared imaginative play, handling conflicts, and practicing reciprocal interaction (Dunn, 2002; Foote and Holmes-Lonergan, 2003; Randell and Peterson, 2009). Given the unique role and lifetime duration of sibling relationships, they often serve as a critical context for social learning; siblings begin as play partners in early childhood and can become a source of support throughout life (Gass et al., 2007; Holmes et al., 2024; Stocker et al., 2020). Such roles might be particularly essential for autistic individuals who show, by definition, persistent challenges with social communication (American Psychiatric Association, 2022). Despite the fact that the definition of autism has evolved over time due to scientific advances and shifting social and cultural influences, difficulties in social interaction have consistently been recognized as a core feature of autism, from early descriptions of the condition to the most recent ones (Happé and Frith, 2020; Jackman and Zwaigenbaum, 2023), and autistic individuals are likely to experience social challenges over the course of their lives (Hendricks and Wehman, 2009; Ozonoff et al., 2010; Zwaigenbaum et al., 2005).

Previous research has shown that having typically developing (TD) siblings is associated with better social functioning in autistic children (Ben-Itzchak et al., 2019, 2016) compared to growing up with no siblings. In a retrospective study, Ben-Itzchak et al. (2016) analyzed records of 112 young autistic children who either had or did not have TD siblings. Comparing the two groups, they found that the group with older TD sibling/s had lower scores in the Autism Diagnostic Observation Scales’ [ADOS-2; (Lord et al., 2012)] Social Affect sub-domain, indicating less severe social difficulties for the group with at least one older sibling than for the group without siblings. This positive sibling effect was specifically pronounced when the TD siblings were older than the autistic siblings. In another study (Ben-Itzchak et al., 2019), this positive effect was replicated with autistic participants (*n* = 150; mean age = 4:0 ± 1:6) who were divided into three equal groups: having no siblings, having older siblings, or having younger siblings. Again, it was found that autistic children with older siblings demonstrated better social functioning than autistic children with no sibling/s, as measured by the ADOS.

Modeling by older siblings and that siblings provide a close, “built-in” social companion to practice social skills were suggested as explanations for this positive effect (Ben-Itzchak et al., 2016, 2019; Rum et al., 2021, 2024b). Using an observational, naturalistic design with a frame-by-frame analysis of sibling interactions at home, in our previous work (Rum et al., 2021), we found an asymmetric role relationship and coordination in the dyadic interaction of autistic children and their older TD siblings: older TD siblings were dominant and led play interactions with their younger, autistic siblings, who were more likely to follow and imitate. These findings of a trend similar to that of dyadic interactions of TD siblings in middle childhood (e.g., Abramovitch et al., 1986) - suggested that modeling by an older TD sibling and a generally collaborative and accepting social environment are possible explanations for the previously found “positive older TD sibling effect” on the social functioning of autistic children. This raises the question of what occurs when autistic children have older *autistic* siblings.

Despite the fact that in many families, there is more than one autistic child (Ozonoff et al., 2024), there is minimal research on autistic sibling dyads and the possible impacts of this relationship on an autistic child. In this case, modeling by an older autistic sibling might lead to more autistic symptoms in the younger sibling and, thus, result in more social-communicational difficulties (i.e., worse social functioning compared to autistic children with TD siblings or with no siblings). However, at the same time, greater companionship might be expected between two autistic siblings under the Double Empathy Problem framework. This theory (Milton, 2012; Milton et al., 2022) suggests that the social-communication difficulties in autism result from mutual and bidirectional difficulties in relationships between autistic and non-autistic people: it is not only that autistic people find it hard to understand typical people and struggle socially but also, non-autistic people struggle to communicate and maintain relationships with autistic social partners. In conceptualizing this model, Milton (2012, Milton et al. 2022) relies on the assumption that it is harder to empathize and thus socially communicate with someone fundamentally different in the way they experience the world than with someone more similar to you. Derived from the theoretical double empathy problem model, recent research has found that autistic people report better connections with other autistic people than they do with non-autistic people (Crompton et al., 2020; Morrison et al., 2020). A recent qualitative study (Crompton et al., 2020) explored the experiences of 12 autistic adults in spending social time with neurotypical and autistic friends and family. The researchers point out that participants frequently described feelings of comfort and ease when spending time with autistic friends and family, and many stated that communication styles were similar among autistic people, and this made interactions more comfortable for them. Considering siblingships within this framework brings up the hypothesis of greater and/or better connection between two autistic siblings compared to mixed siblingship, potentially leading to more social interaction between the siblings that might impact social motivation and practicing of social skills.

The present study aimed to investigate whether findings of a positive effect of older TD siblings on the social functioning of autistic children replicate and whether having older autistic siblings is associated with a similar or perhaps with an opposite pattern. The study’s hypothesis was that by comparing the social functioning of autistic children with no siblings to those of autistic children with older TD siblings, we would replicate previous findings and find that autistic children with TD siblings perform better. Regarding the comparisons between autistic children with older autistic siblings to those with no siblings or with older TD siblings, there was a theoretical basis for hypothesizing in both directions: either a negative effect of a sibling on social functioning (if the autistic sibling is modeling deficits in social communication) or a positive effect (if siblings’ understanding and companionship is higher between two autistic siblings). To the best of our knowledge, this is the first study to examine “the sibling effect” on social functioning in autistic sibling dyads; we thus had no previous empirical literature to rely on in determining a directional hypothesis.

## 2 Materials and methods

### 2.1 Participants

Potential candidates for inclusion in the study were 2,039 autistic children who completed the Autism Diagnostic Observation Scale [ADOS-2; (Lord et al., 2012)] at several centers that provide diagnosis and treatment services and are involved in research in the field of autism affiliated with the Azrieli National Centre for Autism and Neurodevelopment Research (ANCAN). ANCAN is a collaboration between Ben-Gurion University of the Negev and 8 clinical sites throughout Israel (including Soroka University Medical Center; the Autism Child and Family Lab at Hebrew University; the Autism Center/ALUT, Shamir [Assaf Harofeh] Medical Center). All centers are part of the National Autism Database of Israel (Dinstein et al., 2020), which was used to retrieve these previously collected data for secondary analyses, with ethical approval from the Helsinki committees at Soroka University Medical Center (SUMC), Shamir Medical Center, and Leumit Health care Services as well as the internal review board of the Hebrew University. Data for the present retrospective study were retrieved from the database in July 2024. From this initial large cohort, first, participants were removed according to the following exclusion criteria: participants older than 18 years of age; non-Hebrew speakers; participants for whom there were missing data for one or more of the relevant demographic variables or the ADOS scores; participants with no final diagnosis of autism; participants who were not living with their biological parents; participants whose older siblings were reported to be non-TD (with a condition other than autism). After the removal, a cohort of 1,100 children eligible to be included in the present study was scanned to identify the following three groups: (1) autistic children with no siblings (No-Sib; *n* = 146); (2) Autistic children with older TD siblings (Older-TD-Sib; *n* = 300); and (3) Autistic children with older autistic siblings (Older-Autistic-Sib; *n* = 40). We did not include the group of children who had both an older TD sibling and an older autistic sibling. We then aimed to match each child from the Older-Autistic-Sib group to a child from the No-Sib, and a child from the Older-TD-Sib group by sex (required to match exactly), age (up to ±11 months age difference), and cognitive scores (up to ±15 points difference), resulting in 29 triads of three matched participants, i.e., a final cohort of *N* = 87 children; 29 participants in each group. Note that as we aimed to reach a perfect match on sex and keep the matching criteria on age and cognitive scores as detailed above, we could not match all individuals in the Older-Autistic-Sib group with participants from the other two groups. We conducted analysis only on the participants included in the complete matched triads. The age range was 3:0–14:0 years, with a mean age of 5.51 years (*SD* = 2.45); each group included nine girls and 20 boys (see Table 1). All the participants in this study were Israeli Hebrew-speaking children.

**Table 1:** Descriptive statistics of the three matched samples (*N* = 87).

	No-Sib	Older-Autistic-Sib	Older-TD-Sib	Full Sample
Sex, *n* (%)				
Female	9 (31%)	9 (31%)	9 (31%)	27 (31%)
Male	20 (69%)	20 (69%)	20 (69%)	60 (69%)
Age in years	5.47 (2.56)	5.48 (2.56)	5.55 (2.43)	5.51 (2.45)
Cognitive score^1^	77.52 (18.45)	77.72 (19.32)	76.62 (16.03)	77.29 (17.78)
SA score^2^*	7.34 (2.17)	6.34 (2.78)	6.03 (2.07)	6.57 (2.40)

Values are presented as mean (SD) unless otherwise specified; TD = Typically Developing; Mean differences scores were analyzed using repeated measures multivariate analyses of variance (ANOVA).

^1^Standardized score (*M* = 100, SD = 15) based on age-appropriate cognitive assessment (one of the scales for assessing developmental cognitive levels: Bayley; Mullen; WPPSI; Wisc).

^2^SA = Social Affect subscale of ADOS (Autism Diagnostic Observation Schedule).

^*^p < 0.05.

### 2.2 Procedure

All the children included in the cohort had been referred to one of the included centers for a comprehensive assessment of a possible diagnosis of autism. The evaluation included, among other assessments, the collection of demographic data and familial histories from the parents. Information was obtained regarding the siblings of the children who were under evaluation and included the number of siblings, their ages, and their medical and developmental histories. The diagnosis of autism included obtaining the standardized Autism Diagnostic Observation Schedule [ADOS-2; (Lord et al., 2012)], and meeting DSM-V criteria for autism/ASD (American Psychiatric Association, 2020). All the professionals involved in the diagnostic process established reliability in the diagnostic tools as required. Cognitive and developmental abilities (IQ/DQ) were assessed using The Mullen Scales of Early Learning (Mullen, 1995), Bayley Scales of Infant and Toddler Development (3rd & 4th ed.) (Aylward, 2020; Bayley, 2006), Harcourt Assessment (Young, 2006), the Wechsler Preschool and Primary Scale of Intelligence (3rd ed.) (Wechsler, 2002), Wechsler Intelligence Scale for Children IV (WISC-IV) (Wechsler, 2003), according to the child’s age and language level. For all the measures, standard scores were used. This research procedure was approved by each center’s Institutional Review Board (IRB) or ethics committees as required. Since the present study was a retrospective study based on information from the participants’ charts, the IRBs did not require additional parental consent. The 3 groups were then compared on the social affect sub-domain of the ADOS. We further performed an exploratory analysis on another measure of social functioning and overall adaptive functioning based on parental reports. However, tools for collecting the parent reports-based measures varied across centers [most used the Adaptive Behavior Assessment System [ABAS-3, (Harrison and Oakland, 2015)], and one of the centers used the Vineland Adaptive Behavior Scales [VABS, (Sparrow et al., 2016)]. In addition, some data on these measures was missing. The present study’s main focus is on the standard measure of social functioning derived from the ADOS. Below we present this analysis and discuss its main results, as well as the limitations and implications of the additional exploratory analyses.

### 2.3 Measures

#### 2.3.1 Clinician-based social functioning measure: Autism Diagnostic Observation Scales – social affect sub-domain (ADOS-SA)

The Autism Diagnostic Observation Scales [ADOS-2; (Lord et al., 2012)] is a semi-structured assessment schedule designed to assess social and communicative functioning in individuals who may have autism spectrum conditions. The specific module administered depends on the examinee’s age and/or expressive language. A total ADOS algorithm score is used for calculating the total symptom severity score (ADOS calibrated severity scales [CSS]) (Esler et al., 2015; Hus et al., 2014), with higher scores reflecting more severe autism symptoms. This score is composed of two sub-domains: (1) social affect (SA), which is a measure of social functioning, and (2) restricted repetitive behavior (RRB). Internal consistency is high for social affect (*α* = 0.91–0.94) for all modules and lower for RRB (*α* = 0.47–0.65). In the present study, we were interested specifically in social functioning, thus using only the ADOS-SA sub-domain scores. The ADOS-SA score ranges from 0 to 10, with a lower score indicating less severe social-communication symptoms, i.e., better social functioning of the examinee.

#### 2.3.2 Parent reports-based social functioning measure: ABAS/VABS

##### 2.3.2.1 Adaptive Behavior Assessment System (ABAS) social functioning sub-domain

Adaptive Behavior Assessment System [ABAS-3 (Harrison and Oakland, 2015)] is a comprehensive standardized system for assessing adaptive skills. It should be emphasized that the ABAS-3 was not specifically designed to assess individuals on the autism spectrum. The tool consists of five forms that analyze adaptive skills. The forms differ to match different age groups and contexts. Classification and assessment are based on three levels: the General Adaptive Composite (GAC), three adaptive domains (conceptual, social, and practical), and individual adaptive skill areas (mean of 100, SD of 15). Higher scores on the ABAS reflect better functioning. The tool has been found to have excellent internal consistency, with an alpha above 0.9.

##### 2.3.2.2 Vineland Adaptive Behavior Scales (VABS) social functioning sub-domain

The Vineland Adaptive Behavior Scales [VABS-3; (Sparrow et al., 2016)] is a standardized caregiver interview designed to assess adaptive functioning in children from birth to 18 years of age. It includes four sub-domains: Communication, Daily Living Skills, Socialization, and Motor Skills, each of which yields a standard score (mean of 100, SD of 15). The total score is the Adaptive Behavior Composite (mean of 100, SD of 15). Higher scores on the VABS reflect better functioning. Reliability data include internal consistency (0.70–0.97), test–retest (0.70–0.90), and interrater (0.70–0.80) for the parent/caregiver interview forms.

### 2.4 Data analysis

To verify the similarities of the pair-matched groups, age and cognitive ability were compared across the three groups using one-way ANOVAs. Shapiro–Wilk tests were used to confirm the normality of differences between pairs. To examine the differences in the autistic children’s social functioning between the three groups, a one-way repeated measures ANOVA test was used for the ADOS-SA subdomain, followed by three paired t-tests (No-Sib - Older-TD-Sib; No-Sib - Older-Autistic-Sib; Older-TD-Sib - Older-Autistic-Sib), with Bonferroni correction for multiple comparisons. For the additional exploratory analyses on the parent reports-based measure of social functioning, a mixed-effects model was employed instead of traditional repeated measures analyses due to missing data across groups. Statistical analyses were conducted using R software via RStudio interface (RStudio Team, 2024; packages: “tidyverse,” “lme4,” “lmerTest,” “emmeans,” “effectsize”).

## 3 Results

### 3.1 Main analysis – social functioning according to ADOS-SA

The Shapiro–Wilk test was conducted to assess the normality of differences between groups. The results indicated that the differences between the No-Sib and Older-TD-Sib groups were normally distributed (*W* = 0.942, *p* = 0.111). Similarly, the differences between the Older-Autistic-Sib and Older-TD-Sib groups also met the assumption of normality (*W* = 0.977, *p* = 0.749), as did the differences between the No-Sib and Older-Autistic-Sib groups (*W* = 0.942, *p* = 0.112). These non-significant *p*-values (*p* > 0.05) across all comparisons suggest that the data do not significantly deviate from a normal distribution, supporting the appropriateness of parametric statistical analyses. To ensure group equivalence, cognitive scores were compared across the three groups using a one-way ANOVA. Levene’s test indicated that the assumption of homogeneity of variance was met. The analysis revealed no significant differences in cognitive scores between children with older autistic siblings (*M* = 77.72, *SD* = 19.32), children with no siblings (*M* = 77.52, *SD* = 18.45), and children with older TD siblings (*M* = 76.62, *SD* = 16.03), *F*_(2, 56)_ = 0.03, *p* = 0.970, indicating that the groups were well-matched on cognitive functioning.

The effect of group (No-Sib/Older-TD-Sib/Older-Autistic-Sib) on autistic children’s social functioning (ADOS-SA) was compared using a one-way repeated measures ANOVA. Levene’s test indicated that the assumption of homogeneity of variance was met (*p* = 0.101). The analysis revealed a marginally significant main effect of sibling status with a moderate effect size (*F*_(2, 56)_ = 3.09, *p* = 0.053, *ηp*^2^ = 0.10, 95% CI [0.00, 1.00]), which indicated a potential difference between at least two groups. Next, three paired-sample t-tests were performed, with Bonferroni correction for multiple comparisons. Results revealed a significant difference between the SA scores of Older-TD-Sib (*M* = 6.03, *SD* = 2.08) and No-Sib groups (*M* = 7.34, *SD* = 2.18) (difference = 1.31, 95% CI [0.51, 2.11], *t*_(28)_ = 3.35, *p* = 0.002, *adjusted p =* 0.007, *Cohen’s d* = 0.62, 95% CI [0.23, 1.01]), indicating that autistic children with older TD siblings performed better social functioning (had lower SA scores) than autistic children with no siblings; a marginally significant difference between Older-Autistic-Sib (*M* = 6.34*, SD* = 2.78) and No-Sib groups (difference = 1.00, 95% CI [−0.13, 2.13], *t*_(28)_ = −1.8, *p* = 0.082, *adjusted p =* 0.247, *Cohen’s d* = 0.40, 95% CI [0.02, 0.78]) indicating a similar but weaker trend (and non-significant after correction for multiple comparisons), with a smaller effect size for autistic children with older autistic siblings compared with autistic children with no siblings. No significant difference was found between the social functioning of autistic children with older TD or autistic siblings comparing Older-TD-Sib to Older-Autistic-Sib groups resulted in no significant difference between the groups (difference = −0.31, 95% CI [−1.67, 1.05], *t*_(28)_ = −0.46, *p* = 0.647, *Cohen’s d* = −0.13), 95% CI [−0.50, 0.24]. Table 2 summarizes the pairwise comparisons.

**Table 2:** Pairwise comparisons of social affect scores between groups.

Comparison	*t*	*df*	*p*	*p adj* ^1^	Cohen’s d	95% CI for *d*	Mean Diff [95% CI]
No-Sib - Older-TD-Sib	3.35	28	0.002	0.007	0.62	[0.23, 1.01]	1.31 [0.51, 2.11]
No-Sib - Older-Autistic-Sib	1.80	28	0.082	0.247	0.40	[0.02, 0.78]	1.00 [−0.13, 2.13]
Older-TD-Sib - Older-Autistic-Sib	−0.46	28	0.647	1.000	−0.13	[−0.50, 0.24]	−0.31 [−1.67, 1.05]

CI = Confidence Interval.

^1p-values adjusted using Bonferroni correction for multiple comparisons.^

To complete our analysis, we conducted Bayesian analyses to quantify the evidence for group differences (Jarosz and Wiley, 2014; Jeffreys, 1961). The Bayesian ANOVA produced a Bayes Factor (BF₁₀) of 1.27, suggesting weak evidence for overall group differences, meaning the data are only 1.27 times more likely under the model assuming group differences than under the model assuming no differences. However, Bayesian paired t-tests demonstrated strong evidence for the difference between the No-Sib and Older-TD-Sib groups (BF₁₀ = 15.98), indicating the data are almost 16 times more likely under a model assuming group differences than a model assuming no differences. The comparison between the No-Sib and Older-Autistic-Sib groups yielded inconclusive evidence (BF₁₀ = 0.82, BF₀₁ = 1.22). The comparison between the Older-TD-Sib and Older-Autistic-Sib groups indicated moderate evidence for no difference (BF₀₁ = 4.55), meaning the data are about 4.5 times more likely under the model assuming no differences than under the model assuming differences. These results align with the results of our primary analyses.

### 3.2 Additional analysis – a parent report-based measure of social adaptive functioning

For the parent report-based measures of social functioning (ABAS/VINLAND social sub-domains), data were not available for all 87 participants. Data were available for 22 participants in each group (75.9% of the total sample, with missing data varying between matched triads such that only 13 triads had fully complete cases). As this was a secondary analysis of previously collected data, the specific reasons for missing data could not be fully determined retrospectively, though clinical records indicate that in some cases, questionnaires were pending parental completion. To examine potential systematic patterns in the missing parent-reported data, we analyzed differences between participants with and without ABAS/VINLAND data. Missing data were equally distributed across groups (24.1% missing in each group). No significant differences were found in cognitive scores (*p* = 0.900), sex distribution (*p* = 1.000), or SA scores (*p* = 0.438). The balanced pattern of missingness across groups and the absence of differences in key variables suggested that data were missing at random. Given this pattern and the matched nature of our design, we employed linear mixed-effects models instead of traditional repeated measures analyses. This approach was selected as it efficiently handles missing data without requiring complete cases or imputation while maintaining the matched nature of the design through random effects. Participants with missing data were significantly older (*M* = 7.18 years) than those with complete data (*M* = 4.97 years; *p* = 0.004). However, this age difference does not compromise our analyses because our matched-triad design ensures that comparisons are made within age-matched groups, and the mixed-effects model preserves these matched comparisons through random effects, even with missing data. The mixed-effects model, with group as a fixed effect and matching as a random effect, revealed no significant differences in social adaptive functioning across groups, *F*_(2, 44.15)_ = 0.69, *p* = 0.506, *η^2^p* = 0.03, 95% CI [0.00, 1.00]. While parental reports scores of autistic children with no siblings were somewhat higher (*M* = 74.8, *SD* = 17.0, *Mdn* = 72.5) compared to those with older TD siblings (*M* = 71.2, *SD* = 17.0, *Mdn* = 73.0) and those with older autistic siblings [who showed the lowest scores (*M* = 69.6, *SD* = 12.8, *Mdn* = 68.0)], pairwise comparisons using the Tukey method showed no significant differences between groups (no siblings vs. older autistic siblings: *diff* = 5.21, *SE* = 4.55, *p* = 0.492, *δ* = 0.38; no siblings vs. older TD siblings: *diff* = 3.49, *SE* = 4.53, *p* = 0.723, *δ* = 0.25; older autistic vs. older TD siblings: *diff* = −1.72, *SE* = 4.55, *p* = 0.924, *δ* = −0.12). These results indicate that in the present cohort, parent-reported social adaptive functioning was comparable across all three groups, although the small-to-medium effect sizes (δ ranging from −0.12 to 0.38) suggest potential differences that might emerge in larger, more statistically powered samples.

## 4 Discussion

The present study compared social functioning between autistic children with older TD siblings, older autistic siblings, and no siblings. We replicated previous findings indicating a positive effect of having older TD siblings on the social functioning of autistic children (Ben-Itzchak et al., 2019, 2016). In that sense, this study provides additional empirical support for the notion that autistic children benefit from having older sibling/s, perhaps through the opportunity to practice social communication. Interestingly, while autistic children with TD siblings outperformed those with no siblings, they did not outperform those with older autistic siblings. This pattern suggests that contrary to potential concerns about modeling autistic behaviors, having an older autistic sibling was not associated with increased social difficulties. Furthermore, the results implied a trend of positive effect of having an older autistic sibling, similar to that of having an older TD sibling, with a somewhat weaker effect size. In other words, the autistic children with autistic siblings demonstrated an intermediate pattern between the other two groups.

This intermediate pattern could be explained by potentially complex relationships between sibling modeling and sibling companionship on the development of social skills in autistic children and their practice with siblings. It could also be that while the contribution of a TD older sibling is activated by the mechanism of scaffolding and modeling (Rum et al., 2021), the contribution of an autistic sibling is derived from feelings of belonging or ease in communication between the siblings, leading to more comfort and companionship in the relationship (Crompton et al., 2020). Future studies should directly address these questions, for example, by examining measures such as time spent together, imitation, and scaffolding in sibling interactions in autistic sibling dyads, and importantly – by assessing qualitative aspects of sibling relationships such as warmth and closeness, support, and conflict, which could provide deeper insights into the social interaction mechanisms and play (e.g., Rum et al., 2024b). Future research should incorporate direct observations and validated sibling relationship scales.

Notably, in existing research, it is difficult to find the perspective of the autistic sibling, as most studies gather data on TD siblings’ perceptions of sibling relationships with or without autism (Hastings and Petalas, 2014; Rum et al., 2024b; Watson et al., 2021). For TD siblings of autistic children, both negative and positive siblingship experiences were reported (see review: McHale et al., 2016). Our previous work (Rum et al., 2024b) assessed sibling relationship qualities in dyads of TD siblings in comparison to siblingship in which the older sibling is a TD child and the younger sibling is autistic using the Sibling Relationship Questionnaire [SRQ; (Furman and Buhrmester, 1985)]. Despite differences between mothers’ and children’s perspectives in this previous study, both mothers and siblings reported less intimacy and less quarreling between the siblings’ pairs in which one of the siblings is autistic, compared with typical siblingships. These findings might suggest that siblings in mixed-neurotype sibling dyads interact less than TD sibling dyads and thus show less quarreling and report less intimacy. In one exceptional study that included reports of autistic participants on their relationship with an autistic sibling (Tillett et al., 2024), researchers suggested that sibling dyads in which one or both siblings are autistic report less intimacy in the sibling relationship but also seem to experience intimacy and warmth differently than sibling dyads who are both non-autistics. Considering the results of the present study alongside previous findings, the importance of exploring the quality of sibling relationships in autistic sibling dyads is again emphasized. Specifically, it would be interesting to directly examine the hypothesis of better communication and connection in same versus mixed neurotype siblingship as might be predicted by the double empathy problem framework.

The intermediate pattern of implied positive but weaker effect of an older autistic sibling on the social functioning of an autistic child could also potentially be explained by mediating or moderating parenting measures. The experience of raising an older child is likely to have an impact on parenting a second child, and in the case of raising an older autistic child and then a younger autistic child, this impact might be even more significant. Raising a second autistic child poses many challenges, but it could also potentially bring opportunities for parenting that will benefit their second autistic child. For example, parents might be more experienced with practices that support their autistic children, have more knowledge, be already equipped with specific needed support from professionals, or hold attitudes that were shaped by their more experienced parenting – all of which might have a direct and/or indirect impact on the social functioning of the second autistic child in the family. Beyond these parental factors, the observed patterns might also reflect broader shared environmental effects, such as family-wide adaptations in daily routines, home environment organization, and social interaction patterns that evolve after having a first autistic child. These speculative explanations should be directly examined in future research.

Interestingly, both the replicated positive “older TD sibling effect” and the similar trend toward better social functioning in autistic children with older autistic siblings compared to those without siblings were observed in direct behavioral assessments (ADOS) but not in parent-reported measures of social adaptive functioning, where no significant group differences were found. Moreover, descriptive statistics revealed a potentially meaningful pattern: autistic children with no siblings showed somewhat higher parent-reported social adaptive scores (*M* = 74.8, *SD* = 17.0) compared to those with older TD siblings (*M* = 71.2, *SD* = 17.0) and those with older autistic siblings (*M* = 69.6, *SD* = 12.8). The effect size for the comparison between children with no siblings and those with older autistic siblings was small-to-medium (*δ* = 0.38), suggesting a potentially meaningful difference that the current study might have been underpowered to detect statistically. The lack of significant findings should, therefore, be interpreted with caution, as the combination of missing data (data available for only 75.9% of the sample) and relatively small group sizes (*n* = 22 per group) may have limited our ability to detect true group differences. Future research with larger samples and more complete datasets would be valuable to further investigate these potential differences in parent reports on social adaptive functioning, particularly focusing on the trend suggesting potentially better parent-reported outcomes for autistic children with no siblings compared to those with older siblings, which would be in sharp contrast with the results based on the observational measure.

One possible explanation for the discrepancy between the observational and the parent report-based measures of social functioning could be a bias in parent reports of children known as a contrast effect, that is, a tendency to exaggerate differences between siblings. A contrast effect when using parental questionnaires was reported in twin studies exploring genetic and environmental contributions to variation in child temperament or psychopathology (Eaves et al., 2000; Simonoff et al., 1998) and also for non-twin siblings (Saudino, 2003; Saudino et al., 2004). This effect was also suggested as a possible explanation (although not directly examined) in a case where parents reported their autistic children’s empathic abilities as higher for those without siblings compared to those with siblings (Rum et al., 2024a) and when reporting on twins dynamics in comparing TD twin dyads to dyads where one of the twins was a non-TD child (Segal et al., 2025). Similarly, parents might report their autistic children’s social abilities relative to their other children. Thus, those parents who do not have another child have nothing against which to compare to. In the present study, considering previous research and the results from the observational measure-based comparisons, it is possible that a contrast effect encourages parents to underestimate their autistic children’s social functioning when they have siblings. Beyond the contrast effect, these discrepancies might also reflect broader measurement biases, including parents’ different frames of reference when evaluating their child’s social functioning, social desirability effects while reporting their children’s social functioning, and varying expectations based on their children’s daily social contexts and opportunities for comparison. Although these interpretations should be taken cautiously due to the missing data and small sample sizes, the results suggest that future studies may benefit from considering both parental and clinical reports and the potential discrepancies between these, and also taking into account whether a parent has more than one child or not when relying on parental reports in assessing social functioning in autistic children.

The discrepancy between standardized observational measures (ADOS-2) and parent reports highlights methodological considerations beyond potential contrast effects. While the ADOS-2 provides a standardized snapshot of social functioning in a structured setting with unfamiliar adults, parent reports reflect observations of their children’s social behavior across multiple natural contexts over time. Additionally, the ADOS-2 and parent report measures might capture different aspects of social functioning - the ADOS-2 focuses on specific social-communication behaviors during assessment, while parent reports typically encompass broader adaptive social functioning in daily life. These methodological differences in what is being measured (specific behaviors vs. general functioning), how it is measured (structured observation vs. daily observation), and who measures it (trained clinician vs. parent) could contribute to the different patterns observed in our results and should be further addressed in future research.

The present study contributes to the accumulating empirical support for the positive effects of having siblings using objective measures (Ben-Itzchak et al., 2019, 2016). At the same time, the results imply that similar to previous findings (Rum et al., 2024a), parent reports might be influenced by comparative frameworks that could mask these benefits. Future research should employ both objective measures and parent reports, with larger samples, to better understand how sibling and parenting dynamics influence social development in autism and how measurement methods might affect our understanding of these relationships.

The findings of this study should be evaluated in light of its limitations. First, the small sample size did not only limit statistical power but also the possibility of examining the possible role of sex, age, and other demographic or clinical characteristics in the effect of siblings on the social functioning of autistic siblings. Additionally, we did not have data on potential confounding variables such as socioeconomic status, parental stress levels, or intervention history, which might influence both sibling relationships and social functioning outcomes. These factors could be particularly relevant when comparing families with multiple autistic children to those with one or no autistic children, as they might face different challenges and have access to different resources. Second, as this was a secondary analysis of previously collected data, we did not have available data on the quality of the sibling relationship and not on parenting measures that might impact siblings’ dynamics, the autistic children’s social functioning, and/or parental reporting on those. These are important targets of direct examinations in future studies. Notably, the missing data in the parent reports measures limited the statistical power of our additional analyses. In addition, the conclusions from this study cannot be generalized to sibling pairs in which an autistic child is the older sibling and the younger sibling is a TD child. Lastly, autism is a varied spectrum, and we encourage future research to explore specific characteristics, such as similarities and differences between siblings in autism symptoms or co-occurring conditions, and their potential impact on the effect of siblings on an autistic child’s social functioning.

Notwithstanding these limitations and the suggested future direction, the present study has several notable strengths and important implications. To the best of our knowledge, this is the first study to directly examine the effect of having an *autistic* sibling on the social functioning of autistic children. This study thus expands and deepens our understanding of the “older sibling’s positive effect” in autism to include autistic siblings and not only TD siblings. Ultimately, this study contributes to a growing body of literature advocating for the importance of siblings, which are not just a background factor but a significant element in the developmental ecosystem of autistic children, and also in the way parents may perceive their abilities and report on them. The findings of this study suggest that siblings can serve as valuable resources for autistic individuals, whether they demonstrate typical development or share autistic traits and behaviors (and diagnosis) with their siblings.

Beyond theoretical contributions, these findings also have practical implications for clinical practice and family support. First, they suggest that siblings, whether autistic or TD, can be valuable resources for supporting social development in autistic children. This insight could inform family-based interventions by incorporating structured opportunities for sibling interaction and joint activities. Second, the different patterns observed between clinical observations and parent reports highlight the importance of using multiple assessment approaches in clinical practice and considering both standardized evaluations and parents’ perspectives of their children’s social functioning in different contexts. Finally, these results could guide the development of targeted support programs that leverage sibling relationships - whether TD or autistic - to enhance social opportunities and development for autistic children.

## Data Availability

Data cannot be publicly available. Requests to access these datasets should be directed to Azrieli National Centre for Autism and Neurodevelopment Research, Ben Gurion University of the Negev, autism@post.bgu.ac.il.
